# Neural Mechanisms of Inflammation-Induced Fever

**DOI:** 10.1177/1073858418760481

**Published:** 2018-03-20

**Authors:** Anders Blomqvist, David Engblom

**Affiliations:** 1 Department of Clinical and Experimental Medicine, Faculty of Medicine and Health, Linköping University, Linköping, Sweden

**Keywords:** fever, cytokines, prostaglandin E_2_, brain endothelial cells, median preoptic nucleus, EP_3_ receptors

## Abstract

Fever is a common symptom of infectious and inflammatory disease. It is well-established that prostaglandin E_2_ is the final mediator of fever, which by binding to its EP_3_ receptor subtype in the preoptic hypothalamus initiates thermogenesis. Here, we review the different hypotheses on how the presence of peripherally released pyrogenic substances can be signaled to the brain to elicit fever. We conclude that there is unequivocal evidence for a humoral signaling pathway by which proinflammatory cytokines, through their binding to receptors on brain endothelial cells, evoke fever by eliciting prostaglandin E_2_ synthesis in these cells. The evidence for a role for other signaling routes for fever, such as signaling via circumventricular organs and peripheral nerves, as well as transfer into the brain of peripherally synthesized prostaglandin E_2_ are yet far from conclusive. We also review the efferent limb of the pyrogenic pathways. We conclude that it is well established that prostaglandin E_2_ binding in the preoptic hypothalamus produces fever by disinhibition of presympathetic neurons in the brain stem, but there is yet little understanding of the mechanisms by which factors such as nutritional status and ambient temperature shape the response to the peripheral immune challenge.

## Introduction

A little more than 20 years ago, Clifford Saper and Christopher Breder summarized in an authoritative review in *The New England Journal of Medicine* what was known at that time about “The Neurological Basis of Fever” (Saper and Breder 1994). While the critical role of peripherally released cytokines for the febrile response was recognized, it was not clear how these substances could signal to the brain since they could not pass the blood-brain barrier. And although it also was known that prostaglandins were involved in the elaboration of fever, it was not clear where and by which cells the fever-inducing prostaglandins were produced. Furthermore, although it was known that the elevated body temperature was generated by increased energy production and diminished energy loss (by peripheral vasoconstriction), little was known about central neural circuits involved. In this review, we will address our current knowledge of these issues and also point out outstanding questions that deserve further investigation.

Fever is a hallmark of infectious and inflammatory diseases. It is generated by the concerted action of various autonomic responses, such as peripheral vasoconstriction and decreased sweating, reducing heat loss, and shivering, and possibly also non-shivering, thermogenesis. Fever is considered beneficial because an elevated body temperature enhances the activity of the immune cells while at the same time it impairs the replication of many microorganisms (Evans and others 2015; Kluger 1991), although controlled clinical studies of the benefit of fever are lacking (Harden and others 2015). The elevation of the body temperature on immune challenge is a stereotypic response seen in all vertebrates, including poikilotherms, which have been shown to prefer a warmer environment when they have an infection (Boltana and others 2013).

It was demonstrated already at the end of the 19th century that fever required the involvement of the brain (see Atkins 1982). The American pathologist/bacteriologist William H. Welch showed that animals with cervical spinal cord transection did not respond with fever when given an intravenous (i.v.) injection of a pyrogen. It was also understood at that time that the inflammatory process resulted in the release of substances that produced the fever. However, it remained for long unclear how these substances, later named endogenous pyrogens and subsequently identified as cytokines (Dinarello 2015), could influence the brain, since the brain was protected by the blood-brain barrier, described early in the 20th century (Goldman 1913). Nevertheless, injection of endogenous pyrogens into the carotid artery was demonstrated to result in a rapid and strong febrile response, suggesting a direct action on the thermoregulatory center in the brain (King and Wood 1958), and this idea was further supported by the finding that when injected directly into the brain, endogenous pyrogens elicited fever when administered into the anterior hypothalamus/preoptic region, but not when injected into other brain areas (Cooper and others 1967). Based on subsequent observations that prostaglandins of the E-series, when injected into the cerebral ventricles, elicited fever (Milton and Wendlandt 1970), it was further suggested that the endogenous pyrogens acted by releasing prostaglandins (Feldberg and Saxena 1971), an idea that was reinforced by the demonstration that antipyretic drugs like aspirin exerted their mode of action by prostaglandin inhibition (Vane 1971).

## Routes for Immune-to-Brain Signaling in Fever

**Figure 1.**
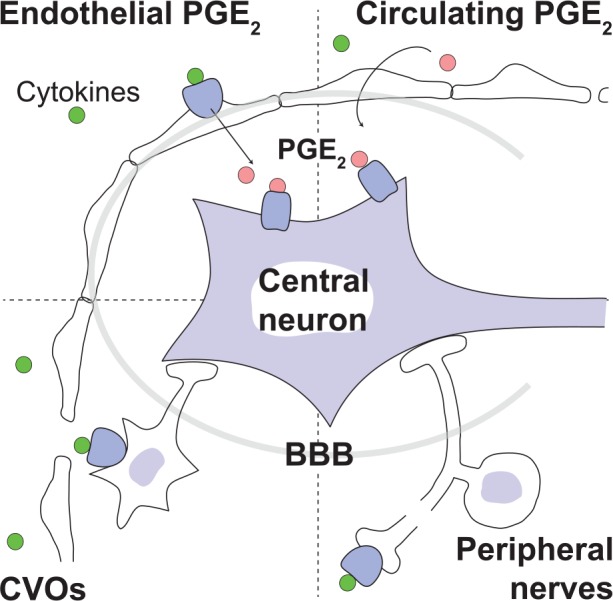
Different suggested routes by which peripherally released inflammatory signals can bypass the blood-brain barrier (BBB) and activate the central nervous system: Peripherally released proinflammatory cytokines (green circles) (i) bind to receptors on cells of brain blood vessels to induce synthesis of prostaglandin E_2_ (PGE_2_; pink circles), which then is transported into the brain parenchyma (upper left); (ii) activate neurons of circumventricular organs (CVOs), which contain fenestrated capillaries (lower left); or (iii) activate peripheral nerves (lower right). (iv) Peripheral inflammation may also release circulating PGE_2_ that enters the brain (upper right).

Over the years, several different hypotheses emerged on how peripheral immune signals could traverse, or circumvent, the blood-brain barrier to influence the brain and elicit fever. These hypotheses include direct pyrogen action on the organum vasculosum of the lamina terminalis, activation of cells in the blood-brain barrier, transfer of blood-borne prostaglandin E_2_ (PGE_2_) into the brain, and activation of peripheral nerves by immune signals (Fig. 1). We will critically examine each of these.

### Role of the Organum Vasculosum of the Lamina Terminalis for Fever

The organum vasculosum of the lamina terminalis (OVLT) belongs to the circumventricular organs (CVOs), which are parts of the brain that lack the normal blood-brain barrier (Siso and others 2010). The capillaries in the CVOs are fenestrated and blood-borne macromolecules can reach the cells within these structures, which hence can monitor essential information of importance for, for example, fluid balance (angiotensin II, natriuretic peptides, osmolarity), metabolic control (amylin, ghrelin, leptin), and reproduction (relaxin). By their efferent projections, neurons in the CVOs can transmit this information to control centers in the hypothalamus and brain stem that serve to maintain homeostasis (Ferguson 2014).

The CVOs were early on suggested as candidates for relaying inflammatory signals to neurons in deep brain structures. In support of this idea, it was shown that the sensory CVOs, which in addition to OVLT include the subfornical organ and the area postrema, express receptors for pathogen associated molecular patterns (PAMPs) and cytokines such as interleukin (IL)-1β, IL-6 and tumor necrosis factor alpha (TNFα) (Ericsson and others 1995; Konsman and others 2004; Laflamme and Rivest 2001; Nadeau and Rivest 1999a, 1999b; Vallieres and Rivest 1997), although it seems as if the receptor expressing cells are endothelial cells and not neurons and preferentially located in the vicinity of the CVOs (Konsman and others 2004; see also Rummel and others 2006). In addition, the sensory CVOs constitutively express microsomal prostaglandin E synthase-1 (mPGES-1) (Eskilsson and others 2014b), a terminal enzyme for PGE_2_ synthesis (Jakobsson and others 1999), implying local PGE_2_ synthesis in these structures. It has also been demonstrated that cells in the sensory CVOs respond with a lower threshold to peripheral immune stimuli than other structures in the brain (Lacroix and Rivest 1997), and that peripheral immune challenge induces cytokine expression in the sensory CVOs (Brochu and others 1999; Nadeau and Rivest 1999b; Quan and others 1999). Furthermore, lesion studies indicate that the area postrema may contribute to IL-1β-induced hypothalamic-pituitary-adrenal axis activation (Lee and others 1998) and to the anorexic response in various disease paradigms, including peripheral cytokine release (Borner and others 2017; Tsai and others 2014).

As for the role of sensory CVOs for fever, the OVLT has been the focus of interest, because of its location adjacent to thermoregulatory neurons in the preoptic hypothalamus. In a study in guinea pigs by Blatteis and collaborators, lesions of the anteroventral third ventricle, including the OVLT, suppressed fever induced by peripheral injection of bacterial wall lipopolysaccharide (LPS), a widely used model for peripheral inflammation (Blatteis and others 1983). However, subsequent studies on animals with lesions of the OVLT have provided contradictory results, with some studies showing attenuated fever, that is, supporting the findings of Blatteis and others (1983), and other studies showing augmented fever or no effect (for a review of the literature, see Romanovsky and others 2003). Because of the small size and position of the OVLT, ablation of the OVLT without damaging adjacent structures has been difficult to achieve, and OVLT lesions have been reported to elicit several acute and chronic effects, which are likely to be due to such additional damage, and which are likely to influence the febrile response. Hence, in one carefully executed study, rats with OVLT lesions were found to exhibit emaciation, hyporeactivity of osmotic stimulation, chronic hypernatremia, and hyperosmolality, and, most important, hyperthermia (Romanovsky and others 2003).

A single study has examined the effect of ablation of the subfornical organ for immune-induced fever (Takahashi and others 1997). In that study, ablation of the subfornical organ attenuated fever elicited by peripheral injection of LPS, whereas ablation of the OVLT or the area postrema had no effect. This study clearly needs to be repeated in independent experiments.

After the recognition of the “side effects” seen following in particular OVLT ablation (Romanovsky and others 2003), interest in the potential role of the CVOs for eliciting fever has faded. However, a recent report comparing the febrile response in mouse lines with different patterns of deletion in brain endothelial cells of MyD88, an adaptor protein for LPS and IL-1β signaling (Xu and others 2000), suggested that fenestrated capillaries in the CVOs were important for fever in response to IL-1β injected into the brain ventricles (Knoll and others 2017). However, apart from differences between the mouse lines regarding MyD88 deletion in fenestrated capillaries, there were several other potential differences between these mouse lines, such as recombination efficacy in the brain endothelium and peripheral immune cells that may have influenced the results. Furthermore, since the IL-1β was given intracerebroventricularly (i.c.v.), it is not clear if the findings are relevant for IL-1-signaling from the periphery to the brain.

### The Blood-Brain Barrier as Transducer of Immune Signals to the Brain

In vitro studies in the late 1980s demonstrated the presence of PGE_2_ production in the brain microvasculature (Bishai and others 1987; Moore and others 1988), but the critical evidence for blood-brain barrier cells as transducers of immune signals to the brain came with the in vivo demonstration of LPS-elicited expression of immunoreactivity for PGE_2_ (Van Dam and others 1993) and of the inducible prostaglandin synthesizing enzyme cyclooxygenase-2 (Cox-2) in these cells (Breder and Saper 1996; Cao and others 1995). However, immediately after the latter discovery the identity of the prostaglandin producing cells became a matter of controversy, since some studies suggested that they were endothelial cells whereas other studies identified them as perivascular cells, immune cells located in the vessel wall on the parenchymal side of the endothelial cells, enveloped by the two sheets of the basal lamina (for a review of the literature, see Rivest 1999). While most investigators now agree on that the vast majority of the brain vascular cells that express Cox-2 in response to moderate and high doses of LPS, independently of route of administration, are endothelial cells, there are still different opinions with regard to the response to IL-1β and to low doses of LPS, with one laboratory reporting that perivascular cells are the main source of PGE_2_ under these conditions (Schiltz and Sawchenko 2002; Serrats and others 2010).

**Figure 2.**
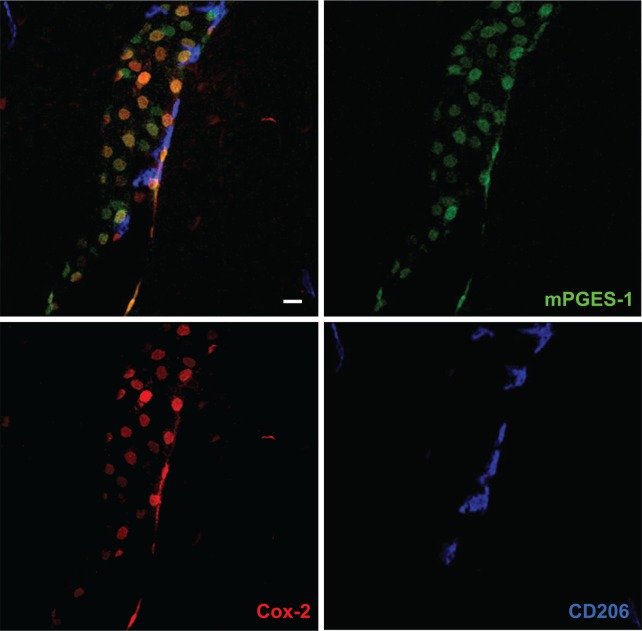
Confocal micrographs of blood vessel in the mouse brain, stained with antibodies against the prostaglandin E_2_ synthesizing enzymes cyclooxygenase-2 (Cox-2) and microsomal prostaglandin E synthase-1 (mPGES-1), and CD206, a macrophage marker expressed by perivascular cells. Upper left panel shows triple labeling for these proteins, and the other panels single labeling for each protein. Note that most cells that express Cox-2 also express mPGES-1 and vice versa. Note also that none of the Cox-2/mPGES-1 expressing cells stain for CD206, implying that this population does not include perivascular cells. Scale bar = 20 µm.

Although the findings demonstrating induced Cox-2 expression in the brain vasculature strongly indicated that blood-brain barrier cells were the site of PGE_2_ production, the definite conclusion was hampered by the lack of evidence that these cells in fact produced PGE_2_, because Cox-2 catalyzes the formation of PGH_2_, which in turn can be transformed into several other prostanoids. The critical and final step came with the demonstration that the inducible terminal PGE_2_ synthase, microsomal prostaglandin E synthase 1 (mPGES-1) (Jakobsson and others 1999), was expressed by brain vascular cells (Ek and others 2001; Inoue and others 2002; Yamagata and others 2001). These studies demonstrated that mPGES-1 was only minimally expressed in the naïve brain of the species examined (rat) but was strongly induced in the brain vasculature following i.v. injection of a low dose IL-1β (Ek and others 2001) and intraperitoneal (i.p.) injection of moderate to high doses of LPS (Inoue and others 2002; Yamagata and others 2001), respectively. The vascular expression of mPGES-1 was localized to cells that expressed IL-1 type 1 receptor (IL-1R1), Cox-2 and an endothelial cell marker (Inoue and others 2002; Yamagata and others 2001; Ek and others 2001; see also Engblom and others 2002b). Subsequent studies, including studies using more natural disease models such as arthritis, burn injury, and inflammation in the paw (Engblom and others 2002a; Ibuki and others 2003; Oka and others 2007; Ozaki-Okayama and others 2004; Rummel and others 2011; Takemiya and others 2010), as well as studies in mice (Eskilsson and others 2014b; Vasilache and others 2015), also found mPGES-1 induction and expression in brain endothelial cells but not in perivascular cells (Fig. 2). There is, to our knowledge, only a single study, performed in rats, that reports mPGES-1 induced expression in perivascular cells (Serrats and others 2010).

**Figure 3.**
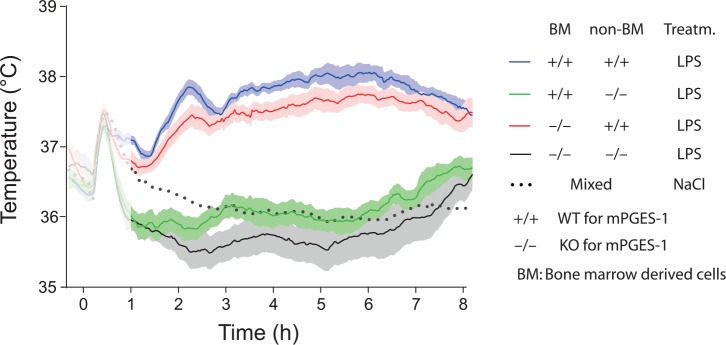
Temperature responses to intraperitoneal injection of bacterial wall lipopolysaccharide (LPS) in wild type (WT) and mPGES-1 knockout (KO) mice that were subjected to whole body irradiation and then transplanted with either WT (+/+) or KO (−/−) bone marrow. Note that WT mice (non-BM +/+) transplanted with WT or KO bone marrow display a prominent febrile response (two top fever curves), whereas KO mice (non-BM −/−) transplanted with WT or KO bone marrow are afebrile (lower fever curves). The initial temperature peak (shadowed) in all groups is handling stress-induced hyperthermia. Replacement of native hematopoietically derived cells was in these experiments about 90% among white blood cells and brain macrophages (perivascular cells), and around 70% among liver (Kupffer cells) and lung macrophages. For the NaCl treated group mean is shown, whereas for the other traces mean and SEM (standard error of the mean) are shown. Adapted from Engström and others (2012).

**Figure 4.**
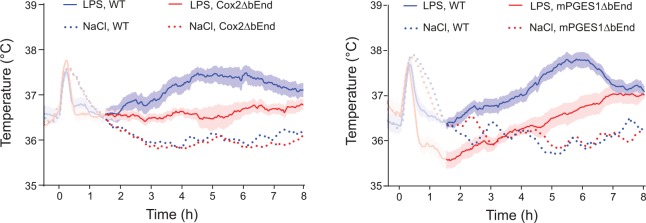
Blunted febrile response to intraperitoneally injected bacterial wall lipopolysaccharide (LPS) in mice with deletion selectively in brain endothelial cells of the prostaglandin E_2_ synthesizing enzymes cyclooxygenase-2 (Cox2ΔbEnd) and microsomal prostaglandin E synthase-1 (mPGES1ΔbEnd). WT, wild type mice. For the NaCl-treated groups mean is shown, whereas for the LPS treated groups mean and SEM (standard error of the mean) are shown. Adapted from Wilhelms and others (2014).

Studies of animals with global deletion of Cox-2 and mPGES-1, which corroborated the idea that these enzymes were critical for fever (Engblom and others 2003; Li and others 1999; Nilsberth and others 2009b; Saha and others 2005), were subsequently followed by functional studies of the role of the prostaglandin synthesis in the brain vasculature for the febrile response. The data obtained so far, show a critical but maybe not exclusive role for the brain endothelial cells. The first functional evidence came in a study by Ching and others (2007), showing that knockdown of the IL-1R1 in brain endothelial cells abolished sickness symptoms (fever and reduced locomotor activity) elicited by i.v. injected IL-1β as well as the induced blood-brain barrier Cox-2 expression and paraventricular hypothalamic Fos expression, hence demonstrating the critical role of endothelial IL-1R1 for these phenomena. Subsequently, Ridder and others (2011) demonstrated that brain endothelial specific deletion of the MAP (mitogen-activated protein) kinase kinase kinase TAK1, which is an important component of IL-1β signaling upstream of the transcription factors nuclear factor–κB (NF-κB) and c-Jun that control Cox-2 gene transcription, resulted in a blunted febrile response to i.v. injected IL-1β. Using chimeric mice that expressed the terminal PGE_2_-synthesizing enzyme mPGES-1 in either hematopoietic cells (including perivascular macrophages) or non-hematopoietic cells (including brain endothelial cells), Engström and coworkers showed that mPGES-1 in non-hematopoietic cells was sufficient for eliciting a febrile response to a peripheral immune challenge, whereas expression of mPGES-1 restricted to hematopoietic cells, including perivascular cells, resulted in ablated fever (Engström and others 2012) (Fig. 3). Finally, Wilhelms and others (2014) provided direct evidence that PGE_2_ synthesis in endothelial cells was critically involved in the febrile response. Using the same tissue specific Cre-mediated recombination as in the study by Ridder and others (2011), Wilhelms and collaborators demonstrated that brain endothelial specific deletion of Cox-2 and mPGES-1 resulted in blunted fever after i.p. injection of IL-1β and LPS (Wilhelms and others 2014) (Fig. 4), whereas deletion in other cell types, such as nerve cells and myeloid cells, has been shown to have no effect (Nilsson and others 2017). The attenuation of fever in the study of Wilhelms and others (2014) was not due to unspecific effects on the inflammatory process, since IL-1β levels in the blood after LPS challenge were unaffected as well as the brain endothelial induction of inflammatory genes (Cxcl10, Ccl2, and Lcn2). This specificity is also demonstrated by the findings that many other symptoms of systemic inflammation are unaffected by the same endothelium-specific manipulations that attenuate fever. Thus inflammation-induced anorexia, inactivity, and hypothalamic-pituitary-adrenal axis activation have been shown to be intact in mice with endothelial manipulations attenuating fever, whereas inflammation-induced place aversion also requires brain endothelial signaling (Fritz and others 2016; Fritz and others 2018; Nilsson and others 2017; Ridder and others 2011; Wilhelms and others 2014). Adding to the finding by Wilhelms and coworkers, it was also demonstrated that deletion of IL-6 receptor alpha (IL-6Rα) and the IL-1R1 on brain endothelial cells attenuated the febrile response to LPS (Eskilsson and others 2014a; Matsuwaki and others 2017; see below). Taken together, the findings from the functional studies using cell-type specific genetic manipulation strongly support a role of the brain endothelium in the generation of fever. Since cell-type specific manipulations are not always 100% specific or selective, it is important to note that the role of the brain endothelium has been shown using two different endothelium specific promoters, that is, the Slco1c-promoter and the Tie2-promoter. It is hence very unlikely that any off-target effects would explain the effects seen in these studies. It is more likely that the importance of the brain endothelium was underestimated, because the Cre-lines used may not target all brain endothelial cells (see, for example, Eskilsson and others 2017).

#### Role of Perivascular Cells for the Febrile Response

While the studies using genetic deletions of inflammatory signaling molecules in endothelial cells hence provide convincing evidence for a critical role of these cells in the febrile response, the contribution of brain perivascular macrophages to fever seems limited. It has been suggested that perivascular cells on the one hand are critical for some aspects of central nervous system activation by IL-1β, but that these cells, on the other hand, subserve an inhibitory action on the PGE_2_ synthesis in endothelial cells and the concomitant acute phase responses elicited by this PGE_2_ production. Using a model in which perivascular cells were ablated by intracerebral injection of chlodronate liposomes (Van Rooijen 1989), Serrats and coworkers reported that such ablation attenuated the cerebral Cox-2 induction as well as the ACTH and corticosterone release in response to i.v. injection of IL-1β, but augmented the same responses following i.v. injection of LPS (Serrats and others 2010). In contrast, the febrile response was intact or moderately enhanced, both to LPS and IL-1β. Thus, although the methodology in this study leaves many open questions regarding the relationship between Cox-2 induction in perivascular cells and the hypothalamic-pituitary-adrenal axis activation, it seems clear that perivascular cells are not important for eliciting the febrile response.

#### Role of IL-1 and IL-6 Signaling Across the Blood-Brain Barrier for the Febrile Response

**Figure 5.**
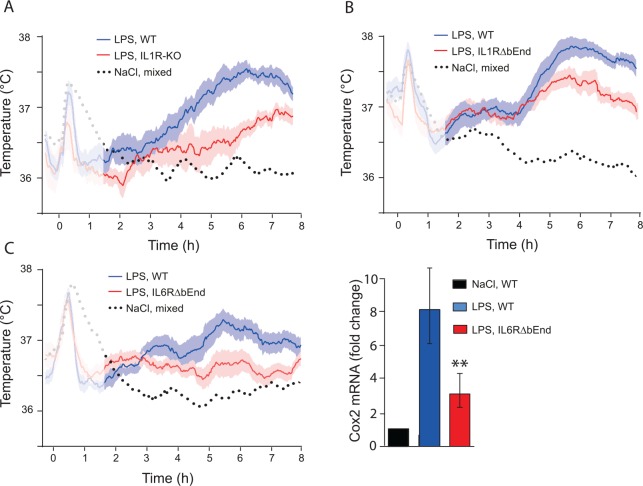
Febrile response to bacterial wall lipopolysaccharide (LPS) in mice with deletion of cytokine receptors. (A) Mice with global deletion of the interleukin-1 type 1 receptor (IL1R-KO) show attenuated fever, however note the late appearing fever in these mice. (B) Attenuated fever, seen after about 5 h, in mice with deletion of the IL-1R1 selectively in brain endothelial cells (IL1RΔbEnd). (C) Attenuated fever in mice with deletion of the interleukin-6 receptor alpha selectively in brain endothelial cells (IL6RΔbEnd) (left). This response was associated with attenuated induction of cyclooxygenase-2 (Cox-2) in the hypothalamus (right). ** indicates *P* < 0.01. Adapted from Matsuwaki and others (2017) and Eskilsson and others (2014).

As described above, IL-1 signaling in brain endothelial cells is critical for the febrile response to exogenously administered IL-1β. However, the contribution of this pathway for the febrile response to a more natural stimulus, such as LPS, is less clear. Thus, mice with deletion of IL-1β not only show intact acute phase responses (Fantuzzi and Dinarello 1996), but even display exaggerated fever (Alheim and others 1997) when immune challenged with LPS. Furthermore, early studies on mice with global deletion of the IL-1R1 and studies on mice that were treated with an IL-1 receptor antagonist reported that these mice displayed intact or only slightly reduced fever in response to LPS (Bluthé and others 2000; Labow and others 1997; Leon and others 1996; Luheshi and others 1996; Smith and Kluger 1992), implying that the IL-1-signaling pathway may not be critical for the febrile response to this stimulus. We recently reexamined this issue, and found that global deletion of IL-1R1, as well as treatment of mice with an IL-1 receptor antagonist, attenuated but did not abolish the febrile response to i.p. injected LPS (Matsuwaki and others 2017). Furthermore, using mice with cell specific deletions of IL-1R1s, we observed attenuation of the febrile response following deletion of IL-1R1 in brain endothelial cells, but not after deletion of this receptor in neural cells or peripheral nerves. In the global knock-out mice, the remaining/attenuated fever was a delayed response, seen from about 5 to 6 hours after the LPS injection, compared with about 3 hours in wild-type mice (Fig. 5). Similar findings were reported by Ching et al. using IL-1R1 knock-down in endothelial cells. While this procedure completely abolished the response to i.v. and i.c.v. injected IL-1β, it only attenuated and delayed the febrile response to i.p. injection of IL-1β (Ching and others 2007). Taken together, these observations seem to suggest that i.p. injection of LPS or IL-1β elicits fever that in part is independent of brain endothelial IL-1R1 signaling.

**Figure 6.**
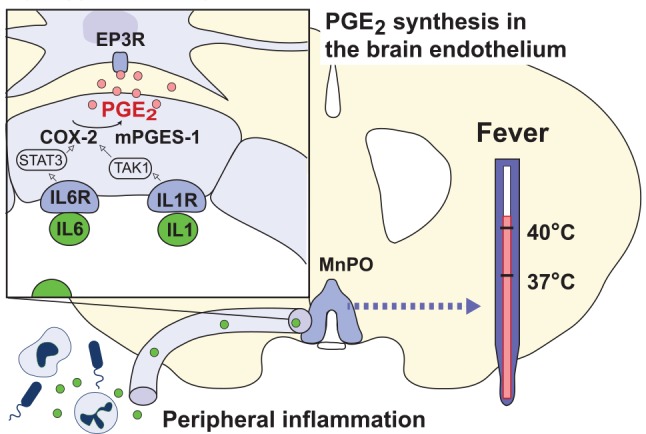
Transduction mechanisms in the blood-brain barrier elicited by peripherally released inflammatory mediators. The cytokines IL-1β and IL-6 (green circles) bind to receptors (IL1R, IL6R) on brain endothelial cells in the preoptic hypothalamus resulting in transcription of cyclooxygenase-2 (COX-2) and microsomal prostaglandin E synthase-1 (mPGES-1) via TAK1 and STAT3, respectively. The subsequent binding of neosynthesized PGE_2_ (pink circles) to PGE_2_ EP_3_ receptor (EP3R) expressing cells in the median preoptic nucleus (MnPO) of the hypothalamus elicits fever.

In contrast to IL-1β, IL-6, which is also released on, for example, LPS challenge, seems to be critical for LPS-induced fever. IL-6 knock-out animals, as well as animals given neutralizing antibodies against IL-6, cannot mount a febrile response (Chai and others 1996; Kozak and others 1998; Rummel and others 2011), in spite of the fact that IL-6 by itself is not or only weakly pyrogenic (LeMay and others 1990; Nilsberth and others 2009a; Rummel and others 2008; Wang and others 1997). The action of IL-6 seems to be exerted via signaling in brain endothelial cells, because mice with endothelial specific deletion of the IL-6Rα showed, as reported above, strongly attenuated fever to peripherally administered LPS, and the same was found for mice with deletion of the downstream signaling molecule STAT3. The IL-6Rα deletion also strongly reduced LPS-induced Cox-2 expression in the brain endothelial cells (Fig. 5) (Eskilsson and others 2014a). An intriguing, yet unanswered question is how the IL-6 signaling pathway interacts with the IL-1-signaling pathway (Fig. 6), and perhaps also with Toll-like receptor 4-signaling, in brain endothelial cells, considering that both cytokines seem to be necessary for the febrile response to LPS. It should be noted that endogenous TNFα, which similar to IL-1β and IL-6 is released by LPS, is a cryogen, although it evokes fever when administered exogenously, probably by releasing other cytokines (Cao and others 1998; Matsuwaki and others 2017).

### A Febrile Response to Blood-Borne PGE_2_?

When mice and rats are given an i.p. injection of LPS, the initial temperature peak induced by the handling stress is followed by a biphasic febrile response (Romanovsky and others 1998). However, if injections instead are given via an indwelling venous catheter during conditions that do not involve handling of the animals, a triphasic febrile response is seen, with the first phase present during the first 60 minutes after injection, and hence obscured by the handling stress that occurs when injections are given i.p. (Romanovsky and others 1998; Rudaya and others 2005). The mechanism behind this first febrile phase has been the subject of much debate and has been suggested to involve activation of peripheral nerves, in particular the vagus nerve, as will be discussed below (see section “Role of Peripheral Nerves for Fever”). However, a prevailing idea, suggested by Romanovsky and collaborators, is that the first phase of fever is elicited by the release of PGE_2_ from lung macrophages into the circulation, and that the blood-borne PGE_2_ enters the brain in the preoptic area, there to elicit the febrile response (Steiner and others 2006). This hypothesis is based mainly on two observations. First, the first phase of fever was found to precede the induction of prostaglandin synthesizing enzymes in the hypothalamus as determined by Western blot, whereas it was concomitant to cyclooxygenase induction seen in peripheral tissues, and in particular that in lung macrophages (Romanovsky and others 2006; Steiner and others 2006). Second, i.v. injection of neutralizing antibodies against PGE_2_ attenuated (but did not abolish) the initial febrile response to peripherally administered LPS (Steiner and others 2006). Both observations need to be interpreted with some caution. Induced synthesis of, for example, Cox-2 in a few but critically located blood vessel (i.e., in the preoptic hypothalamus) may not have been detected by protein analysis of the entire hypothalamus. It has been shown that blood vessels in key autonomic regions display more pronounced expression of IL-1 receptors and downstream intracellular signaling molecules than blood vessels in other regions (Konsman and others 2004). Furthermore, recent work in this laboratory has demonstrated strong Cox-2 mRNA induction in the hypothalamus associated with high levels of PGE_2_ in the cerebral fluid already 30 minutes after i.v. injection of LPS in mice, as well as Cox-2 protein expression in hypothalamic blood vessels (Eskilsson and others, unpublished). As for the experiments with the neutralizing antibodies, it should be noted that the effect was only partial. While this observation could be due to a failure of the antibodies to completely neutralize all circulating PGE_2_, it also opens for the possibility that an additional mechanism for the initiation of fever, independent of circulating PGE_2_, exists. Yet another possibility that cannot be excluded is that a small proportion of the injected antibodies partially penetrated the blood-brain barrier and there neutralized PGE_2_ produced by endothelial cells. By demonstrating that a small amount of antibodies injected i.c.v. did not affect the initial phase of fever, Steiner and others (2006) tried to control for the possibility that minute amounts of the systemically injected antibodies had penetrated the brain and exerted their action in the brain parenchyma. However, this experiment may not be conclusive, because it is not clear that the concentration of antibodies in the extracellular fluid surrounding the EP_3_ receptor expression neurons in the preoptic area was high enough to block locally produced prostaglandins.

**Figure 7.**
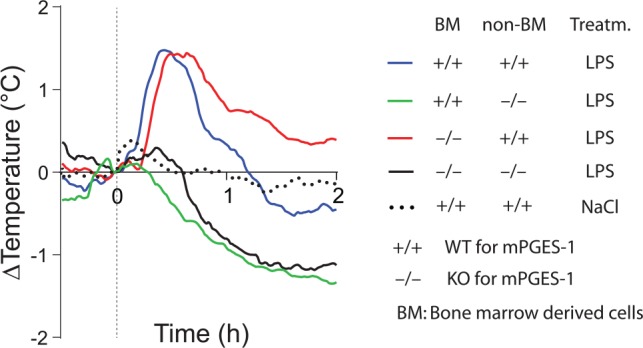
Fever response to intravenously injected lipopolysaccharide (LPS) in whole-body irradiated wild type (WT) and mPGES-1 knockout (KO) mice transplanted with WT (+/+) and KO (−/−) bone marrow. WT mice (non-BM +/+) show a first phase of fever, irrespective of whether they were transplanted with WT or KO bone marrow (upper two traces; cf. the temperature curve for mice injected with saline). In contrast, KO mice (non-BM −/−) transplanted with WT bone marrow instead show a hypothermic response, similar to KO mice transplanted with KO bone marrow (lower two traces). For all traces mean is shown. Dashed vertical line indicates time of injection. For further details of these experiments, see Figure 3. Adapted from Engström and others (2012).

Most important, however, the idea that PGE_2_ produced by peripheral macrophages gives rise to the initial phase of fever has been directly tested and seemed disproved. In the previously mentioned study using mice chimeric for mPGES-1, the terminal isomerase in which absence there is no fever in response to LPS (Engblom and others 2003; Eskilsson and others 2017; Nilsberth and others 2009b), Engström and others (2012) showed that mice with deletion of mPGES-1 in hematopoietic cells displayed a normal first phase of fever, whereas mice that expressed mPGES-1 only in hematopoietic cells showed no fever (but instead hypothermia) (Fig. 7). Notably, the latter mice showed significantly elevated levels of PGE_2_ metabolites in plasma, but not of PGE_2_ in the cerebrospinal fluid (CSF), 40 minutes after LPS injection, implying that the transplanted hematopoietic cells were functional with respect to their prostaglandin producing capacity. Thus, while this experiment confirmed that at least part of the circulating PGE_2_ seen early after LPS injection is of hematopoietic origin, it indicates that this PGE_2_ does not elicit a febrile response, and, furthermore, that it does not seem to enter the brain, at least not to an extent that is reflected in elevated PGE_2_ levels in the CSF. A similar finding was reported in a study on tumor-bearing mice (Ruud and others 2013). In that study high levels of PGE_2_ as well as of PGE_2_ metabolites were recorded in plasma, yet there was no fever and no elevated PGE_2_ levels in the CSF; it should be noted, however, that conditions could be different when elevated PGE_2_ levels occur during inflammatory conditions that could change the permeability of the blood-brain barrier.

### Role of Peripheral Nerves for Fever

The role of peripheral nerves as sensors of peripheral inflammation has been the subject of extensive research, with the vagus nerves being most studied. Early studies using transection of the vagus nerve reported that the vagus nerve was involved in various sickness symptoms such as changes in pain sensitivity, feeding behavior, social exploration, sleep, and stress hormone release (Bluthe and others 1994; Bret-Dibat and others 1995; Gaykema and others 1995; Hansen and Krueger 1997; Kent and others 1996; Luheshi and others 2000; Watkins and others 1994). In support of these observations, the nodose ganglion was shown to express mRNA for the IL-1R1 as well as the EP_3_ receptor (Ek and others 1998; Nakamura and others 2000), the PGE_2_ receptor subtype critical for the febrile response (Ushikubi and others 1998), and i.v. or intraportal injection of IL-1β was shown to increase the discharge activity of vagal afferents (Ek and others 1998; Niijima 1996). The putative downstream signaling mechanisms have been little explored, but analysis of slice preparations of the lower brainstem has shown that PGE_2_ elicits an EP_3_-mediated synaptic depression of vagal synaptic transmission that involves G_i/o_ proteins coupled to adenylyl cyclase (Marty and others 2008).

As for the role of the vagus nerve in fever, the available data are conflicting. Early observations that surgical transection of the vagus nerve attenuated or abolished the febrile response to i.p. injected LPS or IL-1β (Hansen and Krueger 1997; Sehic and Blatteis 1996; Watkins and others 1995) were subsequently questioned (Caldwell and others 1999; Hansen and others 2000; Luheshi and others 2000) because the vagotomy-produced malnutrition may render the animals incapable of mounting a febrile response (Hoffman-Goetz and Kluger 1979; Inoue and others 2008; Krall and others 2010; Shido and others 1989). Also, chemical denervation of visceral afferent fibers with capsaicin was reported to attenuate fever (Szekely and others 1997), but this effect has later been suggested to depend on a non-neural mechanism, such as capsaicin-induced alteration of endotoxin action in the liver (Dogan and others 2004; Petervari and others 2005).

However, the possibility remains that monophasic fever, seen in response to threshold doses of IL-1β or LPS is mediated by the vagus nerve. Thus, it was reported that this response, a brief fever peak of less than 1°C that is seen when the immunogen is given i.v. under conditions not involving handling stress, is abolished following vagotomy also in rats in which malnutrition is prevented (Romanovsky and others 1997a; Romanovsky and others 1997b). It seems to depend on the hepatic branch of the vagus nerve, but not on other vagus branches or on the splanchnic nerve (Dogan and others 2003; Simons and others 1998). Such as role for the hepatic branch of the vagus nerve fits into the suggestion that the first phase of polyphasic fever, which however may not be similar to the monophasic fever (Ootsuka and others 2008), is mediated by activation of the vagus nerve (Blatteis 2007; Li and others 2006; Romanovsky and others 2000). This activation has been ascribed to a complement-induced release from liver Kupffer cells of PGE_2_, synthesized by constitutive cyclooxygenases and hence not requiring de novo protein synthesis. However, this idea has been challenged. Thus, both following subdiaphragmatic and cervical vagotomy (the latter in anesthetized rats), i.v. injection of PGE_2_, believed to mimic its endogenous peripheral release, still elicited fever (Ootsuka and others 2008). Because the fever was abolished by inhibition of the presympathetic neurons in the medullary raphe nuclei, the thermogenic effect of the peripherally administrated PGE_2_ was ascribed to a direct action of PGE_2_ on the brain (see above) not involving the vagus nerve (Ootsuka and others 2008).

In addition to the vagus nerve, somatic afferent fibers have also been suggested to be involved in the febrile response. Similar to the nodose ganglion, dorsal root ganglia have been reported to express IL-1R1 and EP_3_ receptors (Binshtok and others 2008; Nakamura and others 2000), and peripheral nerves have been shown to respond to IL-1β and PGE_2_, although these inflammatory mediators were found to sensitize the nerves to other stimuli rather than to elicit a discharge per se (Binshtok and others 2008; Derow and others 2007). In line with the idea that fine afferent fibers throughout the body sense the internal milieu and through their afferent discharge influence various autonomic relay structures to maintain homeostasis (Craig 2002), it is conceivable that somatic afferent fibers could mediate the febrile response to localized peripheral inflammation. However, in most models used, such as LPS injection into an air pouch or into an artificial subcutaneous chamber there is almost invariably leakage of cytokines, particularly IL-6, from the site of the local inflammation into the circulation (Cartmell and others 2000; Miller and others 1997; Ross and others 2003; Rummel and others 2006; Zhang and others 2008), and systemic treatment with an IL-6 antiserum abolishes the febrile response (Cartmell and others 2000), suggesting that a humoral rather than neural route is responsible for the fever. When a local anesthetic was injected together with LPS into a subcutaneous chamber, fever but not circulating IL-6 levels was attenuated, which was interpreted as evidence that a local neural activation was responsible for the fever (Ross and others 2000). However, as acknowledged by the authors, local anesthetics may interfere with the immune response (Schmidt and others 1997; Sinclair and others 1993) and inhibition of, for example, IL-1β (Sinclair and others 1993), an important co-factor for the pyretic effect of IL-6 (Cartmell and others 2000), may have attenuated the febrile response.

A few studies using localized inflammation have reported that under some conditions no Cox-2 upregulation was seen in the brain despite the presence of fever. Rummel and colleagues injected a low, yet pyretic dose of LPS into an artificial subcutaneously implanted Teflon chamber in guinea pigs, but did not detect any hybridization signal for Cox-2 in the brain (Rummel and others 2005), and Zhang and colleagues, employing casein injection into a subcutaneous air pouch in rats, likewise reported absence of Cox-2 induction in the hypothalamus, as determined by immunoassays and real-time PCR (Zhang and others 2008). These observations hence suggest that even though there is a leakage of IL-6 into the circulation from the local inflammatory site, there is no activation of the central prostaglandin synthesis. While these data are intriguing and may be interpreted as pointing to the presence of a neural afferent pathway for fever, they also suggest that such a pathway might feed into the central thermoregulatory system without involving activation of EP_3_ receptors in the preoptic hypothalamus, that is, in the same way as peripheral thermoreceptors (Blatteis 2007; Morrison 2016). As discussed by Ross and collaborators, strong candidates obviously are cold sensitive fibers. It should be noted that the TRPA1 ion channel, a chemosensor that is activated by noxious cold in rats and mice (Chen and others 2013) and that is expressed on nociceptive afferent fibers (Julius 2013), is activated by LPS, mediating acute neurogenic inflammation and pain (Meseguer and others 2014). With these observations in mind, we recently examined the involvement of TRPA1 in LPS-induced fever. However, TRPA1 KO mice displayed the same febrile response to i.p. LPS as did WT mice (unpublished).

Further support for the role of somatic afferent fibers for fever comes from two studies in which transection of peripheral nerves was reported to attenuate fever. In one of the studies fever elicited by LPS injection into a gingival pouch in the maxilla was shown to be attenuated after transection of all trigeminal nerve branches emerging from the infra-orbital foramen (as it was after local injection of a local anesthetic or a cyclooxygenase inhibitor) (Navarro and others 2006). However, this report lacks confirmation that the surgical procedures, which seem likely to influence the animals’ ability to chew properly, had not affected their body weight and hence their thermoeffector capacity. In the other study, transection of the glossopharyngeal nerve attenuated the febrile response to LPS or IL-1β injected into the soft palate, but not when the pyrogens were injected i.p. (Romeo and others 2001). However, the effect was small, the differences in temperature between the nerve-transected and sham operated groups amounting only to tenths of degrees, and the temperature responses obviously influenced by the general anesthesia during which the injections were performed.

A few studies so far have used genetic deletion techniques to examine the role of cytokine receptors on peripheral nerves for the febrile response. Mice with deletion of IL-6Rα in neural crest derivates that include peripheral nerves, or in vanilloid receptor expressing fine afferents (i.e. nociceptive C-fibers), displayed intact febrile response to i.p. injected LPS (Eskilsson and others 2014a), and the same was found for mice with similar deletions of the IL-1R1 (Matsuwaki and others 2017). In both studies, deletion of IL-6Rα and IL-1R1 in brain endothelial cells attenuated the febrile response (see above). While these data speak against a direct activation of peripheral nerves by peripherally released IL-6 and IL-1β, it should be noted that a neuronal route of immune-to-brain communication might play a role only when high levels of circulating cytokines are absent (Quan 2014; Ross and others 2000; Rummel and others 2005). Because considerable amounts of cytokines are released into the circulation when LPS is injected in the dose used in the two studies reported above (Matsuwaki and others 2017), it is conceivable that a contribution of sensory nerves could have escaped detection.

In sum, the data on the role of peripheral nerves in fever are contradictory. An obvious weakness in all studies is that they examine whether peripheral nerves are necessary for fever, that is, whether interrupting the signaling will attenuate the febrile response. This approach implies, as noted above, that it be difficult to detect if neural signaling exists in parallel with, for example, humoral signaling. Studies in animals with e.g. expression of signaling molecules involved in immune-induced fever specifically on peripheral nerves would help resolve this issue. Such animal models are now becoming available (e.g., Liu and others 2015).

## Central Neurons Critical for the Febrile Response

It is well-established that PGE_2_, through the activation of EP_3_ receptors in the central nervous system, is the final mediator of fever induced by systemic immune challenge with LPS or cytokines (Engblom and others 2003; Lazarus and others 2007; Nilsberth and others 2009b; Saha and others 2005; Ushikubi and others 1998). Although peripheral inflammation results in strong transcriptional upregulation of pyrogenic cytokines not only in the periphery but also in the brain, brain-produced cytokines do not elicit fever in the absence of induced PGE_2_ synthesis (Nilsberth and others 2009b). It was early recognized that the preoptic hypothalamic area played a critical role for eliciting fever (Cooper and others 1967), and this region was subsequently found to be the site that was most sensitive to the pyrogenic action of PGE_2_ (Scammell and others 1996; Scammell and others 1998) and to express high concentration of EP_3_ receptor expressing neurons (Ek and others 2000; Oka and others 2000; Vasilache and others 2007). It is also the site at which local restoration of PGE_2_ production results in a temperature response to LPS in otherwise fever-refractive animals (Eskilsson and others 2017).

**Figure 8.**
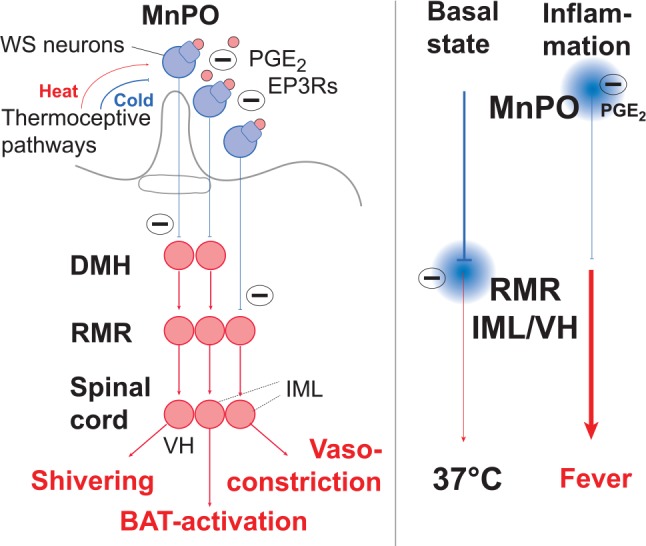
Circuitry of pyrogenic pathways from the preoptic hypothalamus. EP_3_ receptor expressing neurons in the median preoptic nucleus (MnPO) exert tonic inhibition on presympathetic neurons in the rostral medullary raphe nucleus (RMR) as well as on neurons in the dorsomedial hypothalamus (DMH), hence silencing, in the resting state, sympathetic, thermogenic output from the intermediolateral cell column (IML) as well as excitatory output to motorneurons in the ventral horn (VH) of the spinal cord responsible for shivering thermogenesis. On immune-induced PGE_2_ release (pink circles) and binding to the EP_3_ receptors (EP3Rs), the MnPO neurons are silenced, resulting in disinhibition of neurons in DMH and RMR and activation of the thermogenic circuitry. In addition to responding to PGE_2_, EP_3_ expressing neurons are warm-sensitive (WS), and hence activated by heat, inhibiting thermogenesis, and inhibited by cold, promoting thermogenesis. Note that the direct projection from MnPO to RMR controls vasoconstriction, whereas the indirect pathway over the DMH controls shivering and non-shivering (brown adipose tissue [BAT] activation) thermogenesis.

But how does the preoptic hypothalamus generate fever? The prevailing idea is that neurons in the preoptic hypothalamus in the healthy animal provide a tonic inhibitory GABAergic input to thermogenic presympathetic neurons in the rostral medullary raphe nucleus (RMR) of the brain stem, and that the preoptic neurons are silenced on binding of PGE_2_ to their EP_3_ receptors, hence providing a disinhibition of the presympathetic neurons (Fig. 8). This idea is primarily based on the findings by Nakamura and collaborators in rat that (1) EP_3_ receptor expressing neurons in the preoptic region project to the RMR, (2) a large majority of the EP_3_ receptor expressing neurons in the preoptic region co-express transcripts for the GABA-synthesizing enzyme GAD67, and (3) injection of the GABA agonist muscimol in the RMR blocks the thermogenesis evoked by PGE_2_ injection into the preoptic hypothalamic region (Nakamura and others 2002). It is also supported by observations that ablation of the preoptic area or knife cuts caudal to this region generate hyperthermia (Almeida and others 2006; Chen and others 1998; Romanovsky and others 2003), hence suggesting that the preoptic region tonically inhibits caudally located thermogenic neuronal groups. However, as will be further detailed below, this description represents a highly simplified view. Here, it should be noted that only a minority of the preoptic cells that project to the RMR are EP_3_ receptor expressing. Most are not (Nakamura and others 2002) and may instead be excitatory glutamatergic cells (Abbott and others 2016). Furthermore, not all EP_3_ receptors in the preoptic region are connected to inhibitory G-proteins. Analysis of the EP_3_ receptor subtypes in the preoptic regions has shown that a considerable proportion represents a splice variant coupled to stimulatory G-protein (Vasilache and others 2007). Whether some cells exclusively express such receptor variants, or whether the different receptor variants are present on the same cells is not known, although preoptic neurons that are excited by PGE_2_ have been identified (Ranels and Griffin 2003). Finally, little is known about the molecular events downstream of EP_3_ activation in preoptic neurons (Vasilache and others 2013). The idea that a tonic inhibitory activity of these neurons is silenced by PGE_2_ is inferred from the release phenomena observed in their downstream targets on PGE_2_ administration (Nakamura and others 2002; Nakamura and others 2005), and observations of the inhibitory role of these receptors in other cell types (e.g., Breyer and Breyer 2001). As to direct observations so far, recordings from cultured anterior hypothalamic neurons showed that PGE_2_ decreased the firing rate in EP_3_ receptor-positive GABAergic neurons (Tabarean and others 2004). Preoptic neurons that are inhibited by PGE_2_ have been shown also to be warm sensitive (Ranels and Griffin 2003), and warm sensitive neurons are found to be GABAergic and to drive thermogenesis through descending projections (Tan and others 2016). However, it is not clear to what extent the populations of warm sensitive and PGE_2_-responsive neurons overlap.

In addition to the direct projection from the preoptic hypothalamus to the RMR, this structure also receives input from the preoptic hypothalamus via a relay in the dorsomedial hypothalamus (DMH) (Fig. 8), a critical node for various stress induced responses (DiMicco and others 2002), including cold induced thermogenesis (Almeida and others 2006). Whereas the projection to the RMR from the preoptic hypothalamus is inhibitory, the input from the DMH is excitatory, albeit under inhibitory control from the preoptic hypothalamus (Nakamura and others 2005). Retrograde tracing experiments have demonstrated that the preoptic projections to the DMH and the RMR, respectively, largely constitute intercalated but distinct populations (Nakamura and others 2009; Yoshida and others 2009). Furthermore, the projection neurons are found in two distinct nuclear groups in the preoptic hypothalamus, the median preoptic nucleus (MnPO) and the dorsolateral preoptic area (DLPO). Both groups provide tonic inhibitory input to their target neurons in the DMH and RMR, but only the MnPO is responsive to the action of PGE_2_, whereas the DLPO has been suggested to mediate thermosensory regulation in response to changes in skin temperature (Yoshida and others 2009). Data suggests that skin vasoconstriction on skin cooling or PGE_2_ delivery in the preoptic hypothalamus is primarily dependent on the inhibition of the direct projection from the preoptic area to the RMR, whereas thermogenesis through sympathetic activation of brown adipose tissue requires the disinhibition of the projection to the RMR from the DMH (Rathner and others 2008). The latter structure has also been reported to mediate shivering thermogenesis, via its projection to the RMR (Nakamura and Morrison 2011) (Fig. 8).

Nakamura and Morrison, based on studies of cold-induced thermogenic responses as well as the thermogenic responses to local PGE_2_ injections, suggest that the PGE_2_ sensitive neurons that provide tonic inhibition onto the DMH are located in the medial preoptic nucleus (MPO) and not in the MnPO, and that the latter structure provides an ambient cold driven GABAergic inhibition of MPO (Nakamura and Morrison, 2008, 2011). However, as pointed out by Yoshida and others (2009), there is no known projection from the MPO to neither the DMH nor the presympathetic neurons in the RMR, and the dense concentration of EP_3_ receptor expressing neurons in the preoptic hypothalamus does not involve the MPO.

It is important to note that during fever, thermoregulatory threshold changes, including an initial upward shift of the threshold for both cold-defense and thermolytic responses, followed by a widening of the interthreshold temperature zone (Vybiral and others 1987). The increased interthreshold also makes the body temperature less stable, permitting the rapid changes in body temperature than can be seen following i.v. injection of LPS. It is also well-known that anesthesia produces a similar poikilothermic state (Díaz and Becker 2010). This fact should be considered when evaluating some of the core publications on the efferent thermoregulatory pathways, because the findings in these publications were obtained from anesthetized preparations, kept artificially at normal body temperature (e.g., Nakamura and Morrison 2008, 2011; Nakamura and others 2002; Nakamura and others 2005).

The close anatomical relationships between the pathways mediating the pyrogenic response to peripheral inflammation and the thermoregulatory responses to changes in ambient temperature is corresponded by a close functional relationship between these phenomena. It is well known that the febrile response is modulated by the ambient temperature. At low ambient temperatures, rats do not display fever, but hypothermia, when challenged with peripheral injection of LPS, especially when this immunogen is given at high doses (Almeida and others 2006; Romanovsky and others 2005).

The febrile response is also modulated by the nutritional status of the animal: Starvation, which leads to depressed body temperature, strongly attenuates the febrile response and/or produces hypothermia when animals that are kept at subneutral ambient temperature are challenged with LPS (Inoue and others 2008; Krall and others 2010; Shido and others 1989), probably through a leptin dependent mechanism (Inoue and Luheshi 2010; see also Faggioni and others 2000; Steiner and others 2004). Both the influences of ambient temperature and nutritional status on the febrile response may be mediated by the DMH. DMH is critical both for LPS-induced thermoregulation and behavioral cold-seeking in response to sepsis (Almeida and others 2006; Wanner and others 2017), and leptin receptor expressing neurons in DMH have been shown to be activated by cold exposure and to be connected with brown adipose tissue via synaptic connections with the RMR (Zhang and others 2011).

The attenuated fever during starvation does not seem to involve attenuated brain PGE_2_ production, because fasting does not alter the febrigenic signaling from the periphery to the brain, that is important for central PGE_2_ synthesis or PGE_2_ levels in the brain. However, fasting attenuates the response to intracerebrally injected PGE_2_ (Inoue and others 2008). It should also be noted that the mechanism behind immune-induced hypothermia seems to be PGE_2_ independent, probably being mediated by a different prostanoid synthesis pathway (Krall and others 2010; Steiner and others 2009), although this hypothermia also has been suggested to be a consequence of the poikilothermic state and behavioral thermoregulation (Wanner and others 2017). It is conceivable that the mechanism promoting hypothermia coexists with the pyrogenic response, but generally is masked or suppressed by the latter, as seen in mice lacking mPGES-1 (Fig. 7). However, episodes with rapid, active temperature fall can be seen also during normal fever, such as following the initial temperature peak in the 3-phasic fever that occurs after i.v. injection of LPS. Hypothetically, the DMH, or with the DMH connected structures, could serve as an integrator that evaluates the strength of the immune stimulus (i.e., the severity the immune challenge) in relation to the metabolic status and the ambient temperature, and that determines whether the available energy resources are sufficient for eliciting fever, or whether hypothermia will be the more adaptive response (Liu and others 2012).

## Conclusions

Twenty years of research since the review article by Saper and Breder (1994) has yielded deep insight into the mechanism of fever. Yet, several important outstanding questions remain. These relate particularly to the afferent limb of the febrile response. Thus, of the potential pathways for transmitting information about ongoing peripheral inflammation to the brain, pyrogen action on the OVLT, activation of peripheral nerves by immune signals, transport of peripherally synthetized PGE_2_ into the brain, and activation of cells in the blood-brain barrier, only humoral signaling via the blood-brain barrier is supported by unequivocal observations. These observations are obtained in studies in which either cytokine receptors or enzymes for PGE_2_ synthesis were selectively deleted in brain endothelial cells and demonstrate that such deletion attenuates the febrile response to peripherally injected immunogen. However, it should be noted that although fever was attenuated in these studies, fever was never completely abolished, at least not when the immunogen (LPS) was given i.p. Hence, additional pathways exist that are responsible for the residual fever. With regard to studies on the efferent pyrogenic pathways, there is yet little understanding on the mechanisms by which factors such a nutritional status and ambient temperature shape the response to a peripheral immune challenge and how the neuronal circuits that determines whether the response will be fever or hypothermia are organized. The fact that most of the functional studies on the efferent pathways have been performed in anesthetized preparation, in which normal thermoregulation is impaired, is also a caveat in our understanding of these pathways.
